# Algorithmic Compression via Pretrained Neural Networks

**DOI:** 10.3390/e28060596

**Published:** 2026-05-27

**Authors:** Tim Genewein, Jordi Grau-Moya, Li Kevin Wenliang, Laurent Orseau, Marcus Hutter

**Affiliations:** 1 Google DeepMind, London N1C 4DJ, UK; 2 ANU School of Computing, Australian National University, Canberra, ACT 2601, Australia

**Keywords:** algorithmic induction, lossless compression, Bayesian inference, meta-learning, compression and LLMs

## Abstract

The success of large neural networks trained for sequential prediction via log-loss minimization over massive and diverse datasets has sparked debate regarding the fundamental limits of this paradigm. While these models are not explicitly programmed to perform planning and search, their behavior increasingly resembles complex reasoning and adaptive problem-solving. This paper reviews a series of theoretical and empirical works, aiming to bridge the gap between the practical success of LLMs and formal theories of computation and intelligence—that is, algorithmic information theory and Universal Artificial Intelligence. Grounded in the framework of memory-based meta-learning, the main argument is that training sequence models to predict the next token across diverse tasks implicitly meta-trains them to perform algorithmic compression, thereby performing (amortized) Bayesian inference over the task in-context. Consequently, when pretrained on a sufficiently rich data distribution, the resulting neural networks behave as if compressing by inferring the generative algorithm producing the observed data. We discuss recent theoretical and empirical evidence demonstrating that this approach can approximate Solomonoff induction in the theoretical limit, match exact Bayesian inference on complex sources in practice, achieve strong compression on out-of-distribution data, and synthesize complex in-context algorithms like chessboard evaluations. As models become more capable and general, the theoretical understanding through the lens of algorithmic information theory, including hard theoretical limits and how far practical models are from them, becomes increasingly relevant. We thus conclude our paper by outlining a number of open research questions to further bridge the gap from well-understood theory to modern machine learning practice.

## 1. Introduction

The field of Artificial Intelligence (AI) has seen a paradigm shift over the last 15 years, driven by the scaling of neural networks—particularly large language models (LLMs) and multimodal foundation models—and of training datasets to unprecedented sizes. By minimizing log-loss (next-token prediction error) over vast and diverse datasets scraped from the internet, these models exhibit capabilities that increasingly resemble complex reasoning and algorithmic problem-solving. At first glance, the empirical success of these models appears disconnected from rigorous, formal theories of computation and intelligence, such as algorithmic information theory (AIT)—rooted in the foundational work of Solomonoff [1], Kolmogorov, and Chaitin—and Universal Artificial Intelligence (UAI) [2,3]. Specifically, a finite neural sequence predictor trained on human-generated text does not explicitly implement Solomonoff induction [1] or other explicit search-based algorithms common in UAI and traditional AI.

However, a closer examination reveals a profound connection between the practice of log-loss minimization and the principles of algorithmic compression via Bayesian prediction. As established by the framework of memory-based meta-learning [4], training a sequence model to predict the next token under a distribution of training tasks implicitly meta-trains a model to perform Bayesian prediction in-context. That is, when observing a novel sequence at test time, the pretrained model produces outputs that are identical to the Bayesian (posterior) predictive distribution, leading to an adaptive algorithm despite frozen weights—that is, an *amortized* Bayesian predictor. Computationally, this enables rapid, zero-shot and few-shot in-context learning [5].

This paper aims to help bridge the gap between AIT and modern AI practice by reviewing a body of past works from the authors (conducted with many collaborators) that formalize and empirically validate the amortized Bayesian predictor perspective. See Table 1 for an overview of the main works surveyed in this paper. The thread that runs through these works is that log-loss minimization over rich training data is a mechanism to force networks to approximate and amortize the mixture of data generating algorithms—mathematically equivalent to algorithmic compression—which goes far beyond simple “statistical matching”. This perspective also inspired the Hutter Prize for Compression of Human Knowledge [6], where prize money is paid out depending on how much a fixed size of (English) Wikipedia can be compressed. In theory, if models were expressive enough and optimizers strong enough, this idea for obtaining algorithmic compressors could be pushed to train networks that approximate Solomonoff’s universal prediction [7] arbitrarily closely (limited by the available compute budget). In practice, there are hurdles to this, but we review empirical results showing that frozen-parameter neural networks can perfectly match exact Bayesian inference on non-trivial sources [8], that large networks pretrained on text can achieve competitive compression rates on multimodal data [9,10], and that meta-training can give rise to complex in-context algorithms, such as amortized valuation of chess positions and moves [11]—a domain where memorization is notoriously futile.

By synthesizing findings across these works, we highlight how AIT provides the necessary theoretical grounding to shed light on how the remarkable capabilities of modern sequence models arise from the simple objective of next-token prediction. Conversely, these models may be viewed as among the most practical approximation to Solomonoff induction achieved to date. Finally, we discuss the inherent limitations of pure prediction models in interactive agency settings [14,15] and propose future directions for achieving agents that can translate universal prediction into universal action [13].

This paper is dedicated to celebrating the 80th birthday of Prof. Paul Vitányi, whose seminal work with Ming Li on Kolmogorov complexity and AIT [16] has profoundly shaped the field and inspired generations of researchers, as well as much of the research reviewed here. We hope that this review highlights how the foundational ideas of AIT are not only mathematically elegant but also increasingly relevant to understanding modern AI systems, how they work, and the fundamental limitations by which they are governed.

## 2. Background and Notation

Here, we introduce the key concepts and notation used throughout this review, unifying presentation across the different works surveyed.

### 2.1. Sequential Prediction and Compression

Let X be a finite, non-empty alphabet. A string of length *n* is denoted as $x_{1:n}=x_{1}x_{2}\ldots x_{n}\in X^{n}$, with $x_{<t}:=x_{1:t-1}$ denoting the prefix of length t−1, and ϵ the empty string. A sequential predictor is a function ρ that maps any prefix $x_{<t}$ to a probability distribution over the next symbol: $\rho (x_{t}|x_{<t})\in \Delta X$, where ΔX denotes the probability simplex over X. The joint probability of a sequence factorizes as follows:

(1) $$\rho \left(x_{1:n}\right)=\prod_{t=1}^{n}\rho \left(x_{t}|x_{<t}\right).$$

The fundamental equivalence between prediction and lossless compression states that any sequential predictor ρ can be converted into a lossless compressor, e.g., via arithmetic coding [17], achieving an expected code length of

(2) $$H\left(\mu ,\rho \right):=E_{x∼\mu }\left[\sum_{t=1}^{n}-\log_{2}\rho \left(x_{t}|x_{<t}\right)\right]$$

bits, where μ is the true data-generating distribution. For a single sequence, arithmetic coding achieves a code length of $-\log_{2}\rho \left(x_{1:n}\right)+O\left(1\right)$ bits, where the O(1) overhead arises from finite-precision arithmetic and end-of-message signaling. Importantly, *minimizing log-loss (a.k.a. prediction error) is equivalent to maximizing compression*, meaning that the objective used to train modern language models [9] can be viewed as a compression objective (see also the Minimum Description Length (MDL) principle [18,19], Blier and Ollivier [20] for a description-length perspective on deep learning models, and Jiang et al. [21] for a practical demonstration of the prediction–compression equivalence in NLP). Note that the cross-entropy (2) takes on its minimum if ρ→μ, that is, when the predictor matches the true data-generating distribution (on the support of μ), and in which case the expected code length becomes the entropy of that distribution [22].

### 2.2. Bayesian Mixture Predictors

Given a countable (discrete) hypothesis class $M=\{\mu_{1},\mu_{2},\ldots \}$ of sequential predictors and a prior w(μ) over M, the *Bayesian mixture predictor* is

(3) $$\xi \left(x_{1:n}\right)=\sum_{\mu \in M}w\left(\mu \right)\;\mu \left(x_{1:n}\right).$$

The conditional prediction can be written as follows:

(4) $$\xi \left(x_{t}|x_{<t}\right)=\sum_{\mu \in M}w_{t}\left(\mu \right)\;\mu \left(x_{t}|x_{<t}\right),$$

where $w_{t}\left(\mu \right):=w\left(\mu \right)\;\mu \left(x_{<t}\right)/\xi \left(x_{<t}\right)$ are the posterior weights after observing $x_{<t}$.

The Bayesian mixture has fundamental optimality properties: its cumulative log-loss regret relative to the true data-generating distribution $\mu^{*}\in M$ is bounded by $\log_{2}\left(1/w\left(\mu^{*}\right)\right)$, which is finite and only depends on the prior probability assigned to the true model. From this, it follows that the per-step prediction error vanishes: the expected KL divergence between the true conditional and the mixture conditional satisfies

(5) $$\sum_{t=1}^{n}E_{x_{<t}∼\mu^{*}}\;\left[D_{\mathrm{KL}}\;\left(\mu^{*}\left(\cdot |x_{<t}\right)\;∥\;\xi \left(\cdot |x_{<t}\right)\right)\right]\leq \log_{2}\frac{1}{w(\mu^{*})},$$

implying that the average per-step divergence decreases as $O(\log\left(1/w\left(\mu^{*}\right)\right)/n)$, i.e., the Bayesian mixture converges to the true source at a rate inversely proportional to the sequence length [2].

Note that the optimality bound of the Bayesian predictor depends only on the prior probability of the true model. If this prior is uniform, then the bound grows only logarithmically with the number of hypotheses in M. Another interesting choice is to pick a complexity measure C(μ) for hypotheses and assign prior weights according to the (exponential) inverse complexity of the hypotheses: $w\left(\mu \right)=2^{-C(\mu )}$, meaning simpler hypotheses are a priori (exponentially) more likely. Importantly, the Bayesian mixture pays only a one-time, additive penalty proportional to the description length of the true model under the prior—a finite cost regardless of the sequence length. Solomonoff’s predictor (7), which we will discuss shortly, is a special case where M contains all computable hypotheses and *w* is the universal prior, which uses a exponential inverse complexity measure to assign prior weights (over countably infinitely many hypotheses).

### 2.3. Kolmogorov Complexity and Solomonoff Induction

Intuitively, the *Kolmogorov complexity* K(x) of a string *x* measures the length of the simplest program that generates *x*—it captures the inherent information content of *x*, independent of any particular encoding. Formally, K(x) is defined relative to a reference universal Turing machine *U* as follows:

(6) $$K\left(x\right):=\min_{p}\left\{l(p):U(p)=x\right\},$$

where l(p) denotes the length of program *p*. While K(x) is incomputable, it is invariant up to an additive constant across different choices of *U*. Most theoretical results are thus “essentially independent” of the choice of *U* (or the “programming language” of *p*), although this constant can be large for short strings [16] and it can matter in practice.

Solomonoff’s *universal prior* [1] extends the idea that the shortest program for a string captures all algorithmic information, from description to prediction: Solomonoff formulated a prior over all strings produced by computable programs, assigning more weight to simpler strings, i.e., produced by shorter programs—a formal instantiation of Occam’s razor. But instead of only keeping the simplest explanation as a hypothesis, Solomonoff considers all hypotheses that explain the data, but ranks them by their (exponential inverse) complexity—a formalization of Epicurus’s principle of multiple explanations. Using the monotone machine formulation (where U(p)=x∗ denotes that the output of *U* on program *p* begins with string *x*), the universal prior *M* is

(7) $$M\left(x\right):=\sum_{p:\;U(p)=x*}2^{-l(p)},$$

where the sum runs over all programs whose output begins with *x*. Note that $M\left(x\right)>2^{-K(x)}$, which can be easily seen by only considering the shortest program whose output starts with *x* in the sum in (7). Often, this Kolmogorov complexity-based lower bound is tight because shorter programs dominate the sum as the contributions of longer programs vanish exponentially. Solomonoff then uses this prior to solve the induction problem, formulated as general sequential prediction: the goal is to predict the continuation of a sequence with minimal cumulative prediction error (or the fewest mistakes). As shown in [1], this can be solved (axiomatically derived) by sequential Bayesian prediction, where the hypothesis class is all computable programs, and the a priori probability of any program is given by the universal prior.

From the conditional form of the universal predictor $M\left(x_{t}|x_{<t}\right)=M\left(x_{1:t}\right)/M\left(x_{<t}\right)$, it can easily be seen that it can be implemented via sequential Bayesian updating over all computable hypotheses. Solomonoff’s convergence theorem [2,16] guarantees that for any computable distribution μ, the total expected squared Hellinger prediction error is bounded:

(8) $$\sum_{t=1}^{\infty }\sum_{x_{<t}}\mu \left(x_{<t}\right)\;\sum_{a\in X}{\left(\sqrt{M(a|x_{<t})}-\sqrt{\mu (a|x_{<t})}\right)}^{2}\leq K\left(\mu \right)\ln2.$$

That is, the Solomonoff predictor’s total prediction error, summed over all time steps, is bounded by a finite quantity proportional to the Kolmogorov complexity of the true distribution. This is a strong on-average convergence guarantee, although it does not provide worst-case bounds for individual sequences. Similar bounds are also available for the cumulative regret (log-loss of *M* compared to the data-generating distribution μ) and the number of prediction mistakes.

## 3. Theoretical Foundations: Meta-Learning and Bayesian Prediction

The conceptual bridge connecting neural network training to Solomonoff induction is memory-based meta-learning. Traditionally, supervised learning is conceptualized as finding a set of parameters θ that minimize prediction error on a specific task, e.g., a set of sequences from a coin with a particular bias. In meta-learning, or “learning to learn”, the model is trained across a distribution of different tasks or environments (e.g., a distribution of sequences from coins with different biases). This section first presents the theoretical insight showing that this approach yields an amortized Bayesian predictor over the task distribution, and how this necessitates the emergence of rapid in-context learning with frozen model parameters.

### 3.1. Meta-Learning and Bayesian Prediction

Consider a parametric sequential predictor $\pi_{\theta }$ (e.g., a recurrent neural network or Transformer) with parameters θ and access to a memory mechanism (hidden state or attention over context). The *memory-based meta-learning* (MBML) protocol [4] repeatedly performs the following functions:

1. Samples a latent source or task τ from a task distribution ψ(τ);
2. Generates one or more task sequences $x_{1:n}∼\tau$;
3. Updates θ to minimize log-loss over these sequences.

In expectation, this minimizes

(9) $$L\left(\theta \right)=E_{\tau ∼\psi (\tau )}E_{x_{1:n}∼\tau }\left[\sum_{t=1}^{n}-\log\pi_{\theta }\left(x_{t}|x_{<t}\right)\right].$$

The model’s parameters θ are shared across tasks. To predict well, the model must learn to rapidly infer the current task from the observed context $x_{<t}$ without weight modifications—that is, it must learn to perform *in-context learning*. The weights thus converge towards implementing an adaptive algorithm via the network’s activations. As we will show next, to reach optimality w.r.t. minimizing prediction error (log-loss), this algorithm needs to be a Bayesian predictor.

As shown by Ortega et al. [4] and elaborated by Genewein et al. [8], the data-generating process of MBML implicitly defines a weighted mixture over the tasks, where the mixture weights are given by the task distribution ψ, which may be implicit (and of complex algorithmic structure) in some cases. The marginal distribution over sequences is $\xi \left(x_{1:n}\right)=\sum_{\tau }\psi \left(\tau \right)\;p\left(x_{1:n}|\tau \right)$, and the Gibbs inequality guarantees that the unique global minimum of the log-loss objective over all predictors is exactly this mixture ξ. The model meta-trained to optimality $\pi_{\theta^{*}}$ thus matches ξ subject to the assumptions of *realizability* (model expressivity and capacity) and *convergence* (optimization success).The exact match between $\pi_{\theta^{*}}$ and ξ thus holds under ideal conditions; in practice, with finite capacity and optimization gaps, this equality typically remains an approximation; see Section 7 for a discussion on empirical deviations When written in its sequential form, it becomes obvious that this is a sequential Bayesian predictor:

(10) $$\pi_{\theta^{*}}\left(x_{t}|x_{<t}\right)=\xi \left(x_{t}|x_{<t}\right)=\sum_{\tau }\underset{\mathrm{posterior}}{\underset{︸}{\psi (\tau |x_{<t})}}\;p\left(x_{t}|\tau ,x_{<t}\right),$$

where $\psi \left(\tau |x_{<t}\right)\propto \psi \left(\tau \right)\;p\left(x_{<t}|\tau \right)$ is the Bayesian posterior over tasks given the context, and with prior ψ. Thus, the model acts as an *amortized Bayesian predictor*: the computationally expensive or intractable process of the Bayesian update has been distilled into the forward pass of the neural network [12].

Crucially, the set of hypotheses (or tasks) of the amortized predictor is defined by the meta-training data distribution (as well as the model’s expressivity, and the convergence of the learning process). If the pretraining dataset consists of varied, algorithmically structured data (e.g., code, mathematics, logical puzzles, or human language), the model is forced to internalize complex algorithms into its hypothesis space in order to predict/compress well algorithmically.

### 3.2. In-Context Learning as a Consequence of Bayes-Optimality

The meta-learning perspective also provides an explanation of the computational mechanism underlying in-context learning (ICL) [5]: the hallmark feature of any Bayesian predictor is that it (on average) adapts most rapidly—that is, most sample-efficiently—to any task from the task distribution. In fact, this is how Bayes optimality is defined—in terms of the lowest average cumulative regret (or similar bounds on the prediction error or the number of errors above a certain magnitude). Since individual tasks under the mixture can be algorithmically complex, this view is perfectly compatible with neural networks exhibiting complex algorithms in particular in-context adaptation settings, such as approximating gradient-descent-based linear regression [23,24], as long as these algorithms can be expressed by the model and are effective predictors for one or more of the tasks in the pretraining mixture. Mechanistic investigations have revealed increasingly sophisticated optimization algorithms inside Transformers: von Oswald et al. [25] showed that trained models can implement mesa-optimization procedures resembling momentum and preconditioned gradient descent, going well beyond simple linear regression. Indeed, Akyürek et al. [24] showed that the learning algorithms implemented in-context by Transformers converge to Bayesian estimators as the model capacity increases, providing a direct bridge between the gradient descent and Bayesian interpretations of ICL. This principle extends beyond supervised learning: Laskin et al. [26] demonstrated *Algorithm Distillation*, where a Transformer trained on multi-episodic RL histories learns to perform in-context reinforcement learning, distilling the improvement operator of algorithms like A2C into its forward pass without any explicit RL objective at test time. Lampinen et al. [27] further argued that any setting in which context reduces prediction loss constitutes a form of in-context learning, extending the meta-learning view to encompass the full breadth of LLM capabilities, including instruction-following and role-playing.

It is worth noting that the theoretical prediction of Bayesian behavior of large pretrained models has been challenged empirically. Falck et al. [28] examined whether ICL in large language models satisfies the martingale property—a necessary condition for Bayesian inference on exchangeable data—and found systematic violations in state-of-the-art models. These deviations do not strictly invalidate the meta-learning framework, which predicts Bayesian behavior only at the optimum of the meta-training objective; rather, they highlight the approximation gap between the theoretical ideal and practical finite-capacity models—an important distinction to which we return in Section 7.

Additionally, Lampinen et al. [29] provided empirical evidence that language models generalize differently—and often more broadly—from information acquired in-context compared to information acquired through fine-tuning. This observation is consistent with the amortized Bayesian perspective: in-context adaptation leverages the full posterior machinery of the pretrained mixture, whereas fine-tuning modifies the prior (and members of the hypothesis set) itself and may overfit to a narrow task distribution.

Under this perspective, prompting or *prompt-tuning* can be viewed as the attempt to efficiently steer an amortized Bayesian predictor by exploiting (or even abusing) its in-context learning mechanism [5,30]. More precisely, the goal of prompting is to find a prompt (typically a prompt prefix) that, when consumed by the model, imbues the internal representation of sufficient statistics with maximum information about some desired target task (for example, think of manipulating the counters of heads and tails for a beta-Bernoulli predictor to 70 and 30, in order to minimize subsequent prediction error on a sequence drawn from a coin with bias of 0.7). Genewein et al. [5] investigated theoretical conditions for settings where optimal prompting is and is not possible. The crucial object to study turned out to be the relation between the pretraining task distribution (giving rise to the Bayesian mixture) and the distribution of target tasks. If there is only a single target task $\tau^{*}$ and it has positive support under the pretraining distribution ($\psi (\tau^{*})>0$), then there always exists a prompt (prefix) $s_{1:L}$ such that the Bayesian posterior concentrates on $\tau^{*}$: $\psi \left(\tau |s_{1:L}\right)\approx \delta \left(\tau =\tau^{*}\right)$. Conversely, Genewein et al. [5] also identified where no prompt in theory can concentrate the posterior sufficiently. A straightforward case is the situation where the target task is a truly novel task outside the support of the data-generating distribution ($\psi (\tau^{*})=0$). More subtle is the case when the target task distribution is a mixture over two or more tasks: even if all of these tasks are in the support of the pretraining distribution (they have non-zero mass under the Bayesian prior), for most likelihood models and priors there is no sequence that could concentrate the posterior over multiple modes (to capture the target mixture), as the Bayesian posterior increasingly concentrates on a single mode in most Bayesian models. Wenliang et al. [30] provided empirical and theoretical analysis of when and why prompting and interpreting optimal prompts is difficult (even under an exact Bayesian predictor on relatively simple data distributions), identifying settings where the geometry of the posterior landscape prevents intuitive prompt-based steering. The question of whether effective optimal prompting schemes always exist for universal prediction (Solomonoff induction) is an open problem (see Genewein et al. [5]).

## 4. Empirical Verification

Having established that memory-based meta-learning yields amortized Bayesian predictors in theory, a natural question is whether this correspondence holds in practice. We now summarize works where the strategy is to verify the equivalence in settings where the true Bayes-optimal predictor is computationally tractable, allowing exact comparison.

### 4.1. Binary i.i.d. Sources and Bandit Tasks

Early work by Mikulik et al. [12] demonstrated that LSTMs, after being meta-trained on simple i.i.d. sources of coin-flip sequences, perfectly match predictions of the exact Bayesian predictor on novel coin-flip sequences. Beyond that, the work also showed that internal states of the network can be matched to the corresponding states of an exact Bayesian predictor that track sufficient statistics (counting the number of observed heads and tails, essentially). Beyond prediction tasks, Mikulik et al. [12] also showed that amortized Bayes-optimal decision-makers can also be obtained via meta-training (on simple bandit problems). While this early work established the principle, the environments considered were relatively simple.

### 4.2. Piecewise Stationary Sources

Subsequent research investigated more complex, non-stationary data streams. Genewein et al. [8] explored sequence prediction tasks generated by piecewise stationary Bernoulli sources with unobserved switching points. This setup is challenging because the predictor must simultaneously infer the current segment’s statistics, detect switching points, and maintain running sufficient statistics for all possible segment lengths and switching points—all purely from the observed binary stream.

The work investigated several switching-point distributions, including the Partition Tree Weighting (PTW) prior (hierarchical partitioning of segments) and LIN prior (considering all switching points). The exact Bayes-optimal strategy for the PTW source is given by the PTW algorithm [31]. Remarkably, LSTMs and Transformers meta-trained purely via log-loss minimization on sequences drawn from this distribution learned to implement an amortized version of PTW. The frozen neural networks closely matched the predictive performance of the exact algorithm (with cumulative regret differences in the order of $10^{-2}$ nats for sequences of length 256) and exhibited the characteristic, rapid belief resets immediately following a source switch. Furthermore, models acquired the corresponding inductive biases: a PTW-trained model performs optimally on PTW data but suboptimally on LIN data, mirroring the exact algorithms.

### 4.3. Variable-Order Markov Sources

**Figure 1:**
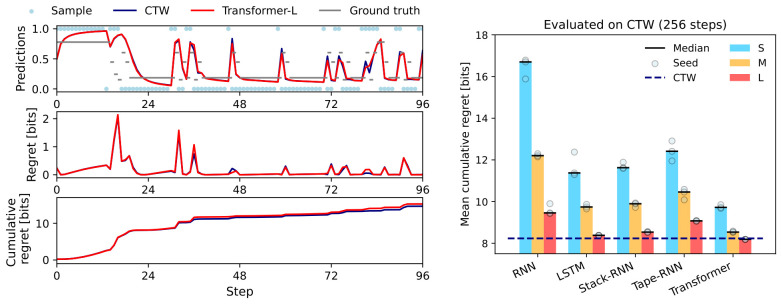
Neural networks were pretrained on sequences drawn from a distribution over variable-order Markov processes with binary alphabet. The figure shows evaluations on data from the same distribution (Grau-Moya et al. [7], Figure 2). **Left:** Single-trajectory predictions on a k-order Markov process drawn from the CTW prior. The meta-trained Transformer closely tracks the exact Bayes-optimal predictor (CTW), including rapid adaptation to changes in the underlying state of the Markov process (ground-truth emission probabilities shown via gray dashes; emitted samples in light blue). Lower panels show per-step and cumulative regret (i.e., prediction error relative to the ground-truth emission probabilities). **Right:** Mean cumulative regret over 6 k sequences (length 256, max. CTW tree depth 24) for different trained networks (3 seeds) and sizes (S, M, L). Larger models perform better for all architectures, and the Transformer-L and LSTM-L match the optimal CTW predictor.

A next step towards “more universal” hypothesis classes is to consider all variable-order Markov processes (VOMS). The exact Bayesian solution for this hypothesis class is the Context Tree Weighting (CTW) algorithm [3,32]. Grau-Moya et al. [7] showed that LSTMs and Transformers, trained on data generated by such processes, reliably converge to Bayes-optimal performance. The results mirror those for piecewise stationary sources: large Transformers and LSTMs match CTW’s performance in terms of cumulative regret, and the predictions overlap nearly perfectly even on individual trajectories (see Figure 1).

## 5. Towards Universal Prediction via Meta-Learning

The empirical results of the previous section confirm that meta-training on tractable hypothesis classes yields predictors that match exact Bayesian solutions. The connection to algorithmic compression, however, requires making the step to a universal computational hypothesis class. This raises a question: can the task distribution ψ be chosen such that the resulting amortized Bayesian predictor is Solomonoff’s induction, which uses Solomonoff’s prior to quantify the a priori probabilities of hypotheses?

Grau-Moya et al. [7] explored this question theoretically and empirically by exploring meta-training on a corpus of sequences sampled by feeding random uniform bit streams (programs) into a (monotone) universal Turing machine (UTM). The resulting program outputs (strings) are generated with probability $2^{-l(p)}$, where *p* is the program. The marginal distribution over outputs *x* is exactly the Solomonoff prior $M\left(x\right):=\sum_{p:U(p)=x*}2^{-l(p)}$ [1]. Following the memory-based meta-learning framework [4], minimizing log-loss over such a dataset amortizes the universal predictor, distilling approximate Solomonoff induction into the forward pass of the network.

To make this idea computationally feasible and address non-halting programs (which lead to semi-measures), Grau-Moya et al. [7] restricted programs to length ≤L and execution steps ≤s, and they removed programs that did not produce an output after *s* steps (which, strictly speaking, corresponds to a normalized Solomonoff prior; see paper for details). The resulting approximation to Solomonoff’s prior was proven to converge to *M* in the limit s,L→∞. Neural networks meta-trained on these datasets thus approximate Solomonoff induction with increasing fidelity for increased *s* and *L*, as well as increasingly expressive architectures (larger models).

However, a major practical hurdle is that sampling uniform random programs from a UTM predominantly generates simple or sparse outputs that lead to low sample efficiency for generating datasets to train models on, and the resulting strings differ significantly from structured data like human language. Grau-Moya et al. [7] addressed this by proving that the sampling distribution over programs does not need to be uniform, introducing the *generalized Solomonoff semi-measure*:

(11) $$M_{U}^{Q}\left(x\right):=\sum_{q:U(q)=x*}Q\left(q\right),$$

where *Q* is a near-arbitrary prior over programs. Convergence to the universal prior is preserved under distributions *Q* that significantly deviate from random uniform, and it may have all kinds of short- or long-range statistical correlations, as long as *Q* has positive support for any finite string and has finite entropy:

**Theorem 1**  (Universality of generalized Solomonoff semi-measures [7]). *$M_{U}^{Q}\left(x\right)$ is strongly universal, provided Q is a computable measure such that Q(q)>0 for all finite strings q, and $Q(q_{1:n})\to 0$ as n→∞. That is, there exists a universal monotone TM V such that $M_{U}^{Q}\left(x\right)=M_{V}\left(x\right)$ for all x*.

This result is of profound importance: it establishes that one can bias the program distribution *Q* towards “interesting” or human-aligned structures without losing the theoretical convergence guarantees towards universal prediction.

Putting together all of these pieces, Grau-Moya et al. [7] constructed a computable approximation of *M* (from which “interesting” samples can efficiently be drawn) by bounding the program length l(q)≤L, target sequence length *n*, and computational steps *s* as follows:

(12) $$M_{s,L,n}^{Q}\left(x\right):=\sum_{q\in {\left\{0,1\right\}}^{\leq L}:U^{s}\left(q\right)=x*}Q\left(q\right)$$

and collecting a finite data set $D^{J}=\left(x^{1},\ldots ,x^{J}\right)$ of *J* samples drawn from $M_{s,L,n}^{Q}$ for meta-training neural predictors. Crucially, for scaling in practice, choosing a prior *Q* biased towards structured syntax (e.g., enforcing balanced brackets for a simple programming language) mitigates the sparse output problem of uniform program sampling. Grau-Moya et al. [7] reported that a simple second-order Markov model for sampling programs could increase the yield of non-trivial data generators by a factor of 137, compared to random uniform sampling, without impacting the universality guarantees. Empirically, the paper demonstrates that pretraining on such data leads to positive compression transfer to algorithmic tasks across the Chomsky hierarchy (such as binary modular arithmetic, or reversing a string; see Delétang et al. [33]) and VOMS data, for which CTW constitutes the exact Bayesian predictor. On a small scale, this supports the idea that training on universal algorithmic data gives rise to a general amortized predictor.

From an algorithmic information-theoretic perspective, this provides arguably the strongest justification for modern LLM pretraining. Instead of worrying about the choice of UTM or designing complex synthetic program generators, collecting vast quantities of diverse, algorithmically dense data (such as code, math, and literature) and minimizing next-token prediction error can be seen as training an amortized Bayesian predictor on a highly biased but approximately universal distribution (with better approximation error the more diverse the underlying data generators are). In the limit of capacity and data, this approach converges towards a universal predictor capable of executing general in-context algorithms.

In practice, even very large Transformers are still bounded (e.g., by a fixed context-window) and are not algorithmically universal [33]. Similarly, even very vast datasets of human-generated data do not cover the outputs of all computable programs. As a result, an approximation gap will persist and its impact can be seen by decomposing the prediction error as follows [34]:

(13) $$\underset{\mathrm{total}\;\mathrm{loss}}{\underset{︸}{E_{\mu }\left[-\log\pi_{\theta }\left(x_{n}|x_{<n}\right)\right]}}=\underset{\mathrm{entropy}}{\underset{︸}{H(\mu )}}+\underset{\mathrm{model}\;\mathrm{class}\;\mathrm{regret}}{\underset{︸}{D_{\mathrm{KL}}\left(\mu ∥M\right)}}+\underset{\mathrm{approximation}\;\mathrm{gap}}{\underset{︸}{D_{\mathrm{KL}}\left(M∥\pi_{\theta }\right)}},$$

where μ is the true data-generating process and *M* is the optimal Bayesian predictor (the Solomonoff prior). The middle term is the theoretical regret characterizing the difference between the true source and the universal prior (with an upper bound that depends on the Kolmogorov complexity of the environment [2]; see Section 2.2). The third term captures the gap between the exact universal Bayesian predictor and its neural approximation. Crucially, as the network’s capacity and expressivity increase, this approximation gap shrinks, and the models consistently surpass context-dependent coding bounds, showing non-trivial compression.

## 6. Empirical Evidence at Scale

One of the main insights of the previous section was a sketch for a formal justification of pretraining large models on large complex datasets from a universal prediction argument. The question that remains is whether this argument is vacuous or not in practice. We have already discussed works that empirically verify that meta-training converges exactly to a Bayesian predictor in non-trivial settings in Section 4. Nonetheless, these settings are still far from LLM scale. Since exact Bayesian solutions are intractable at that scale, we have to turn to indirect measures instead of comparing against exact solutions. In particular, we investigate the general (algorithmic) compression performance of neural nets compared to strong general-purpose compression algorithms, such as gZip or LZMA, or domain-specific compressors, like PNG and FLAC.

### 6.1. Language Modeling Is Compression

Delétang et al. [9] conducted a systematic study demonstrating that large language models (LLMs), exclusively trained on text, can act as highly effective, general-purpose compressors across multiple modalities. When applied to image (ImageNet 64 × 64 patches) and audio (LibriSpeech) data without any domain-specific fine-tuning, frozen-parameter LLMs like Chinchilla 70B outperformed both general-purpose and specialized codecs like gZip, LZMA, PNG, and FLAC in terms of raw compression ratios, compressing image byte sequences to 48.0% (compared to PNG’s 61.7%) and audio to 21.0% (compared to FLAC’s 30.3%) (see Table 2). This cross-modal transfer suggests that the models have acquired the ability to detect (via in-context learning) and exploit general-purpose algorithmic patterns to compactly represent data outside their original training distribution. These results are in line with the view that minimizing log-loss over a training dataset produced from highly diverse algorithmic sources leads towards a more and more general—and in the limit universal—predictor/compressor.

These findings are closely related to the broader program of Shaw et al. [34], who provided a systematic analysis of how optimizing description length objectives drives neural models to learn algorithmic structures that minimize Kolmogorov complexity. Their work establishes formal connections between the loss landscape of neural sequence models and information-theoretic quantities from AIT, including an explicit decomposition of prediction error into irreducible entropy, model class regret, and approximation gap—a decomposition that we have shown in Section 5. From a Minimum Description Length (MDL) perspective, Bornschein et al. [35] provided a detailed empirical analysis of how neural sequence models behave under prequential MDL evaluation. Their work showed that the rate at which neural networks discover and exploit the underlying data structure—measured by the transition from initial high code lengths to near-optimal compression—closely mirrors the learning curves predicted by Bayesian mixture theory. This provides a view of log-loss training via SGD as implicitly performing a form of model selection consistent with the MDL principle, where simpler models (shorter programs) are favored early in training and more complex structures are incorporated as evidence accumulates. The meta-learning theory presented earlier refers only to the final trained network as an amortized Bayesian predictor, but it makes no predictions about training dynamics.

Recent work by Shinnick et al. [36] showed that Transformers pretrained on procedural data develop modular internal structures for algorithmic reasoning, providing mechanistic evidence for the compression-based perspective. Complementary evidence comes from Li et al. [37], who found that a GPT model trained purely on sequences of Othello game moves develops an emergent internal representation of the board state—a form of world model that arises solely from the pressure to predict the next move accurately.

### 6.2. Compression Perspective on Scaling Laws

An important insight from the compression viewpoint is a nuanced take on scaling laws [9]. While large models (Chinchilla 70B) were shown to achieve lower raw compression rates of the data compared to standard algorithms, the *adjusted* compression rate—which also takes into account the model size—reveals a U-shaped curve. Any fixed-size dataset has a corresponding optimal model size that best trades off a two-part code description length of the data to compress. Scaling beyond that size degrades the total compression because the increased model parameters overwhelm the savings on the data. In the work of Delétang et al. [9], where the size of the test data to compress is always 1 GB, using pretrained billion-parameter models to do so always constitutes a suboptimal trade-off and worse adjusted compression rates compared to standard algorithms. This was further investigated by Heurtel-Depeiges et al. [10], who showed that using smaller neural networks can lead to competitive compression performance even when comparing parameter-count-adjusted compression rates. In particular, relatively small models (e.g., up to 20 M parameters) can beat general-purpose and domain-specific compression algorithms in terms of adjusted compression rates (on 1 GB of data). However, the big caveat is that these results only hold for “in-modality” test data, e.g., if the pretraining mixture is text and ImageNet patches, the strong compression performance applies to text and image patches from other sources (e.g., CelebA), but not on audio data. This is a crucial qualitative difference from the behavior of very large pretrained models in the work of Delétang et al. [9], which showed strong compression transfer to modalities unseen during training for large models. This transfer must arise somewhere in the regime between the largest models in the work of Heurtel-Depeiges et al. [10] (20 M parameters) and the smallest models that showed transfer in the work of Delétang et al. [9] (billion-parameter scale), and it may also be affected by the particular composition of the training data (which is larger and from more diverse sources, but only from one modality, for pretrained LLMs, compared to the models in the work of Heurtel-Depeiges et al. [10] that were trained on smaller but multimodal datasets in some experiments). Studying this threshold regime, where general algorithmic compression abilities start to emerge, and how this is affected by model size, architecture, and pretraining mix, is an interesting open question.

### 6.3. From Passive Compression to Amortized Planning

A striking example of emergent in-context algorithmic capabilities is provided by Ruoss et al. [11], who meta-trained a Transformer purely via log-loss on sequences of moves from 10 million chess games (corresponding to 530 million unique board states). Each board state (or each move in each board state, depending on the setting) was annotated with an (action) value from a very strong chess engine (Stockfish 16). The model after training, without any explicit search algorithm (like Monte Carlo tree search) or reinforcement learning at test time, was able to mimic the value estimate of the complex chess engine. When used in a policy, it exhibited strong chess play and the ability to solve difficult chess puzzles, which requires accurate calculation of value estimates (see Table 3 for concrete results). Importantly, the results cannot be explained by forms of memorization, as most board states at test time (and all puzzles) were unseen at training, thanks to the high branching factor of chess, where most board states (taken from actual chess games) are unique, even in a collection of millions of games (very early opening states appear in many games, of course). The model essentially amortized the look-ahead computation of the chess engine into its forward pass, confirming that log-loss prediction over complex structural data forces the network to implicitly implement the data’s generative algorithm. It is unclear to what degree the trained model internally implements forms of search and heuristics, or a kind of “world model” for planning. While the specific models of Ruoss et al. [11] have not been fully analyzed, recent mechanistic interpretability work on other chess-playing networks, such as Leela Chess Zero, has begun to address this. Jenner et al. [38] found evidence of learned internal look-ahead, with intermediate layers representing future board states rather than merely the current position. Cruz [39] further investigated and characterized these look-ahead mechanisms. The findings suggest that the amortized forward pass does not simply memorize state–value associations but implements a non-trivial computational process related to search. However, fully characterizing these internal algorithms remains an open challenge for mechanistic interpretability. It should also be noted that despite using models with up to 270 million parameters, the gap to the chess engine could not be fully closed, suggesting that a larger model and/or more training data would be needed (although it cannot be fully ruled out that the Transformer architecture poses fundamental algorithmic limitations in some cases). Concurrent with Ruoss et al. [11], Monroe and Chalmers [40] published a detailed tech report on the Leela Chess Zero community’s “ChessFormer”—a Transformer-based architecture that uses clever domain-specific adaptations (including a custom encoding called smolgen). Their comparison shows that ChessFormers comparable in size outperform the vanilla Transformers of Ruoss et al. [11] while requiring fewer FLOPS, confirming the amortized planning capabilities that were independently explored by the open-source chess engine community.

These results, together with the emergent world models observed in Othello [37] and the in-context simplicity bias documented by Deora et al. [41] and Elmoznino et al. [42], suggest that log-loss minimization over structured data induces not merely statistical pattern matching but genuine algorithmic compression. The models demonstrably internalize the computational structure of their training data.

Perhaps the most ambitious demonstration of amortized agency to date is the Adaptive Agent (AdA) of Adaptive Agent Team et al. [43], who meta-trained a large-scale Transformer-based agent on an open-ended 3D environment with a vast set of procedurally generated (multi-agent) tasks. At test time, for novel tasks, AdA exhibits human-timescale adaptation: given a novel, never-before-seen task specified only by a reward signal, the agent adapts its behavior within a handful of episodes—far faster than conventional RL agents, which require millions of environment steps, and on par with human adaption rates. This rapid adaptation arises from the same memory-based meta-learning principle discussed in Section 3: the agent’s parameters encode a prior over task-specific policies, and the in-context memory (episodic observations and rewards) serves as a conditioning context that drives posterior-like updates over the agent’s internal policy.

## 7. Limitations and the Gap to Agency

While the empirical success of large pretrained models establishes them as powerful amortized predictors, a critical limitation remains when attempting to use these models as interactive agents. The theory of universal prediction (Solomonoff induction) is a foundational component of Universal Artificial Intelligence (AIXI) [2,3], but prediction alone is insufficient for optimal action in an interactive environment.

### 7.1. The Inference and Support Gap

**Figure 2:**
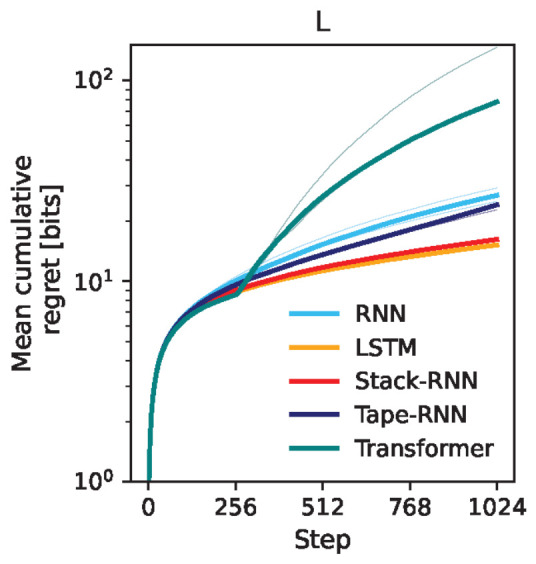
Length generalization to OOD sequence lengths (1024 steps, trained on 256; Grau-Moya et al. [7], Figure 2). LSTMs degrade gracefully, while Transformers fail (likely due to out-of-distribution positional encodings). See Figure 1 for in-distribution performance of the models. Thin lines are individual training runs, while thick lines are the median over 3 seeds.

Several theoretical caveats limit the direct application of Solomonoff induction to modern neural networks—most notably, the meta-distribution gap. The strict equivalence between meta-trained networks and Bayesian predictors requires that the test-time data lies within the support of the pretraining distribution. A fundamental limitation of the Bayesian theory is that it provides no guarantees for out-of-distribution inputs: once we leave the training distribution, amortized predictors may deviate arbitrarily from the exact Bayesian predictor. This is illustrated in Figure 2, which shows length generalization on the VOMS task from Figure 1. When evaluated on sequences of 1024 steps (4× the training length of 256), the amortized neural predictors’ length generalization performance is quite different: LSTMs degrade relatively gracefully, while Transformers fail catastrophically—likely due to out-of-distribution positional encodings. Note that the exact Bayesian predictor for this case, CTW, generalizes perfectly to arbitrary sequence lengths. This highlights that even when a neural network perfectly matches the Bayesian predictor in-distribution, its OOD behavior is governed by architectural inductive biases rather than Bayesian theory.

For internet-trained LLMs, the support of the pretraining distribution is vast but bounded, and characterizing it is challenging: defining a “task” for internet-scale text remains elusive, making it difficult to demarcate the boundaries of in-distribution data. Chan et al. [44] showed that specific distributional properties of the pretraining data—burstiness and label diversity—are necessary for in-context learning to emerge at all, suggesting that the composition of the meta-training distribution plays a critical role beyond mere scale. In practice, large Transformers generalize remarkably well to diverse sources. Under the meta-learning view, this success could stem from a sufficiently broad pretraining distribution combined with high model capacity and powerful optimization, effectively treating novel tasks as “in-distribution”. Alternatively, we might operate in an out-of-distribution regime where the architecture’s strong inductive biases are the driver of generalization. Disentangling these two effects remains an open research question.

Furthermore, the (strict) meta-learning argument depends strongly on realizability and optimization convergence. We can formalize the gap between the theoretical Bayesian predictor and the neural approximation as the *amortization gap*:

(14) $$\Delta_{\mathrm{amort}}\left(x_{<t}\right):=D_{\mathrm{KL}}\;\left(\xi \left(\cdot |x_{<t}\right)\;∥\;\pi_{\theta }\left(\cdot |x_{<t}\right)\right),$$

which measures the excess prediction error of the neural approximation $\pi_{\theta }$ relative to the exact Bayesian mixture ξ at each context. This gap arises from three sources: finite model capacity (the model class may not contain ξ), finite training data (the empirical meta-training distribution differs from ψ), and optimization error (the training procedure may not find the global optimum). The total excess loss is bounded but may be substantial in practice, as evidenced by the systematic deviations from Bayesian behavior observed by Falck et al. [28]. Expanding the theory to give practical bounds for the case of limited realizability and convergence is an important direction for future research, which will require extending the Bayesian view of meta-learning.

### 7.2. Causal Delusions and the Knowing–Doing Gap

Another challenge in extending amortized prediction to interactive agency stems from the causal structure of the data distribution. Standard pretraining via log-loss amortizes the process of Bayesian conditioning (passive observation). In this regime, the network learns to update its internal state based on observed sequences $x_{<t}$, approximating the conditional probability $P(x_{t}|x_{<t})$. Just as Solomonoff induction itself is mathematically passive and does not model interventions, standard log-loss minimization on offline datasets provides no inherent incentive for exploration.

However, an interactive agent does not merely observe the environment; it intervenes. In causal terms, taking an action corresponds to the *do-operator* [14,45], which requires updating beliefs differently than standard conditioning, particularly in the presence of unobserved latent variables (confounders).

When a model is trained to predict the next token on offline, third-person interaction data (e.g., expert demonstrations), it amortizes the belief updates of a passive observer. At test time, if the model’s actions are merely sampled from this predictive distribution, it treats its own actions as observed data points rather than interventions. This mismatch manifests as *causal delusions* [14]: the model fails to distinguish between correlations observed in the expert data and the causal effects of its own actions. This phenomenon is closely related to the causal confusion problem identified by de Haan et al. [46] in the imitation learning literature, where behavioral cloning models exploit spurious correlations in expert demonstrations rather than learning the true causal policy. Consequently, the model might attempt to replicate the state distribution of the expert without executing the underlying causal chain, leading to compounding errors. To amortize the do-operator update and achieve robust agency, the learning algorithm must involve online interaction or active learning, where the model can observe the consequences of its interventions.

While the causal delusion framework provides a clean theoretical account, the empirical picture reveals a broader constellation of failure modes. An early illustration came from DeepMind’s GATO [47]—a single generalist Transformer trained via supervised imitation across over 600 tasks, including Atari games, robotic manipulation, and dialogue. Despite impressive breadth, GATO consistently underperformed specialist systems and, crucially, could not exceed expert-level behavior—a ceiling inherent to offline imitation learning. More recent benchmarks have confirmed and extended this pattern. Ruoss et al. [15] introduced LMAct, a benchmark for in-context imitation learning with up to one million tokens of expert demonstrations across six interactive tasks (tic-tac-toe, chess, crosswords, Atari, grid-world navigation, and simulated locomotion). Frontier LLMs (at the time) were shown to possess factual knowledge of optimal strategies—for instance, when asked directly, they can articulate how to play tic-tac-toe optimally—yet consistently failed to translate this knowledge into effective decision-making when deployed as interactive agents. Moreover, providing more expert demonstrations often yielded no improvement or even degraded performance, suggesting that the difficulty lies not in information access but in the ability to act on the basis of the observed demonstrations. Notably, the paper frames this as a *knowing–doing gap* rather than attributing it to causal delusions specifically, although it acknowledges the theoretical relevance of delusions in partially observable settings [14]. The BALROG benchmark [48] further corroborates these findings across six challenging game environments (including NetHack, Crafter, and TextWorld), showing that even the best models achieved only modest game progression and that visual observations often hurt rather than helped performance. Importantly, these failures have proven resistant to the rapid scaling of model capabilities. Despite advances in reasoning (chain-of-thought prompting, reasoning-specialized models like o1) and alignment (RLHF), interactive task performance remains brittle as of (early) 2026.

On the theoretical side, recent work by Ortega [49] proposes a principled resolution by extending Solomonoff induction from passive strings to interactive transcripts. The key insight is an epistemic one: in a joint universal mixture over computable generators of action-observation histories, the agent’s own actions must be treated as *interventions* (choices) rather than evidence, so that posterior weights update only through the world’s responses. Under this first-person discipline—which directly addresses the causal delusion pathology—behavior follows from sampling the mixture’s action channel, effectively treating agency as pattern completion under interventions. Ortega proved a finite cumulative divergence bound between the agent’s actions and counterfactual target actions, implying that large deviations occur only finitely often. Notably, this framework subsumes reward maximization as a special case: rewards become one kind of observation among many—alongside demonstrations, language, and feedback—rather than the primitive definition of purpose. While this theoretical proposal has not yet been empirically validated, it offers a concrete path toward reconciling universal prediction with universal agency. In principle, it seems straightforwardly doable to extend the meta-learning protocol accordingly—the problem in practice is that training would require interventions (and an environment or simulator that supports these). Methods like reinforcement learning from human feedback (RLHF) or self-play are empirical attempts to correct the mismatch between the pretrained predictive prior and the desired active policy, but bridging the causal gap in a theoretically principled and scalable manner is still an open challenge.

### 7.3. Self-AIXI

Bridging the gap from prediction to optimal action requires moving beyond passive modeling. A theoretically sound framework for this is proposed by Catt et al. [13] with *Self-Predictive Universal AI (Self-AIXI)*. While the standard AIXI agent [2,3] derives its policy from exhaustive planning (search) inside a Solomonoff environment model ξ, Self-AIXI shifts the computational burden from planning to learning by self-predicting its own future action stream.

Specifically, in addition to holding a universal mixture over environments ξ, the agent maintains a Bayesian mixture over a class of policies *P*:

(15) $$\zeta \left(a_{t}|h_{<t}\right):=\sum_{\pi \in P}\omega \left(\pi |h_{<t}\right)\pi \left(a_{t}|h_{<t}\right)$$

where $\omega (\pi |h_{<t})$ is the posterior probability over policies. Self-AIXI then evaluates the expected action-value function $Q_{\xi }^{\zeta }$ over both mixtures:

(16) $$Q_{\xi }^{\zeta }\left(h_{<t},a_{t}\right):=\sum_{\pi \in P}\omega \left(\pi |h_{<t}\right)\sum_{\nu \in M}w\left(\nu |h_{<t}\right)Q_{\nu }^{\pi }\left(h_{<t},a_{t}\right)$$

The Self-AIXI policy $\pi_{S}$ is defined by a single greedy step over these on-policy estimates: $\pi_{S}\left(h_{<t}\right):=\arg\max_{a_{t}}Q_{\xi }^{\zeta }\left(h_{<t},a_{t}\right)$. By performing Bayesian updates on its own generated action data, the policy mixture absorbs the computational effort of policy improvement. Catt et al. [13] proved that this self-predictive distillation converges to AIXI asymptotically in expectation for any environment μ∈M:

(17) $$E_{\mu }^{\pi_{S}}\left[V_{\xi }^{\pi^{*}}\left(h_{<t}\right)-V_{\xi }^{\pi_{S}}\left(h_{<t}\right)\right]\to 0\;\mathrm{as}\;t\to \infty$$

effectively demonstrating that distillation serves as a robust theoretical alternative to traditional amortized search.

## 8. Open Questions and Future Directions

The interpretation of modern sequence models as amortized algorithmic compressors and Bayesian predictors opens several promising avenues for future research:

- **Practical Bounds on Algorithmic Complexity and Approximation Gap:** While we have theoretical bounds on the approximation gap (Section 5), calculating or tightly bounding the Kolmogorov complexity of real-world datasets remains impossible. Developing practical Minimum Description Length (MDL) approximations [35], verifiable information distances for large models [34], and establishing tight bounds on the approximation gap under finite parameters, finite datasets, and imperfect optimization convergence are crucial for grounding the theory in realistic settings and establishing practical benchmarks for universal prediction.
- **Mechanistic Interpretability of Internalized Algorithms:** Wenliang et al. [30] provided initial insights into the internal representations of in-context Bayesian inference, while Shinnick et al. [36] showed that Transformers trained on procedural data develop modular internal structures. Recent work on chess-playing neural networks, such as Leela Chess Zero, has revealed a form of learned look-ahead, with intermediate layers encoding future board states [38,39]. However, the full mechanisms by which models implement search-free algorithms—such as the Chess Transformer’s amortized value computation—remain largely unknown. Understanding these internal structures and the algorithmic limits implied by certain architectures remains an important open problem. Equally important is disentangling whether generalization on novel tasks stems from a sufficiently broad pretraining distribution (rendering a large set of tasks as “in-distribution”) or from architectural inductive biases enabling true out-of-distribution generalization.
- **Scaling Laws for Algorithmic Priors:** Current scaling laws characterize log-loss as a function of parameter count and dataset size. However, how does the algorithmic diversity of the dataset influence the learned prior? Investigating the sample complexity of learning universal predictors and establishing scaling laws with respect to task diversity is an important open question.
- **Efficient Prompting of Universal Predictors:** An interesting open theoretical question is whether an approximation to a universal predictor like Solomonoff induction can be efficiently steered. Genewein et al. [5] asked whether Solomonoff’s predictor can be computationally prompted using prefixes of (relatively) short length to guarantee optimality on arbitrary downstream target tasks. While (gradient-based) prompt-optimization techniques worked well in practice for amortized Bayesian predictors on simple algorithmic data sources, giving a theoretical answer to the question is an open problem.
- **From Universal Prediction to Universal Agency:** As discussed in Section 7, translating a universal predictor into an optimal agent requires overcoming causal confounding and characterizing the meta-distribution gap. Key challenges include developing meta-learning objectives that encourage causal models [50], amortized do-operations, or structural mechanisms for active decision-making [13].

### Additional Related Work

The connection between in-context learning and Bayesian inference has been explored from several complementary angles. Xie et al. [51] formalized ICL as implicit Bayesian inference by modeling pretraining data as a mixture of hidden Markov models. Concurrently, von Oswald et al. [23] and Akyürek et al. [24] provided mechanistic evidence that Transformer self-attention layers can implement gradient descent and ridge regression, converging to Bayesian estimators with sufficient model capacity. Garg et al. [52] systematically characterized the function classes that Transformers learn in-context, including linear functions, sparse linear functions, and decision trees. Kirsch et al. [53] explicitly meta-trained Transformers for general-purpose in-context learning across diverse task families, demonstrating that a single model can adapt to classification, regression, and sequence prediction tasks purely through its context window. Chan et al. [44] showed that specific distributional properties of the pretraining data—burstiness and diversity—are necessary for ICL to emerge, connecting the data-generating process to the meta-learning framework. Yadlowsky et al. [54] further showed that pretraining data mixtures enable narrow model selection capabilities: Transformers trained on diverse function classes can implicitly select the appropriate model class from context, although this capability remains limited to classes seen during training. In a broad unifying perspective, Lampinen et al. [27] argued that any setting in which context reduces prediction loss constitutes a form of in-context learning, encompassing few-shot classification, instruction-following, and role-playing under a single meta-learning umbrella. Coda-Forno et al. [55] provided evidence that large language models exhibit meta-in-context learning—the ability to adapt not just their predictions but their learning algorithm itself based on the structure of the context, suggesting that LLMs have internalized a hierarchy of learning strategies. Mirchandani et al. [56] showed that LLMs can serve as general pattern machines, capable of completing complex token sequences, spatial patterns, and robotic trajectories zero-shot, providing further evidence for the view that these models have internalized general-purpose algorithmic primitives through pretraining.

The amortized Bayesian perspective adopted in this review is part of a broader literature on amortized inference, where computationally expensive posterior computations are distilled into neural network forward passes. Early work by Ravi and Beatson [57] formalized amortized Bayesian meta-learning from a variational inference standpoint, while Grant et al. [58] showed that gradient-based meta-learning (MAML) can be interpreted as hierarchical Bayesian inference. The Prior-Fitted Networks (PFN) framework of Müller et al. [59] and its application to tabular classification [60] demonstrated that Transformers pretrained on data sampled from a prior can perform approximate Bayesian prediction in a single forward pass—a concrete instantiation of the amortization principle. Similarly, BayesFlow [61] uses normalizing flows for simulation-based amortized inference across scientific models. Recent work by Reuter et al. [62] extends the amortized Bayesian paradigm to full posterior inference for generalized linear models and latent factor models.

The compression perspective on neural sequence models has a rich history. Blier and Ollivier [20] showed that prequential (online) coding leads to shorter description lengths for deep learning models than two-part codes, providing an MDL perspective on neural network generalization. Jiang et al. [21] demonstrated competitive text classification using only off-the-shelf compressors and normalized compression distance, illustrating the prediction–compression equivalence in a practical NLP setting. Most directly relevant to the present review, Wan and Mei [63] recently formalized the argument that next-token prediction in LLMs implements approximate Solomonoff induction, providing a complementary formal treatment to the meta-learning perspective presented here.

## 9. Conclusions

In this review, we have synthesized a growing body of literature bridging algorithmic information theory and modern machine learning practice. The central message is that large pretrained sequence models are best understood not as mere engines for statistical matching but as amortized Bayesian predictors, performing algorithmic prediction or compression, and approximating towards the theoretical ideal of Solomonoff induction (although the approximation gap is still large today and will always remain non-zero). We traced this perspective from its theoretical foundations, to exact empirical verification for i.i.d., piecewise-stationary, and variable-order Markov sources. Additionally, we have discussed the theoretical extension to universal prediction, which is the formal piece that connects modern ML practice with AIT and Solomonoff induction.

However, practical complications and open problems remain. We have highlighted that successful amortization is sensitive to the meta-distribution gap, realizability constraints, and standard optimization bottlenecks. More fundamentally, pure prediction is insufficient for interactive agency, requiring a transition from amortized conditioning to amortized intervention—a challenge that demands active learning or potentially new meta-learning paradigms.

Ultimately, the convergence of deep learning and algorithmic information theory serves as a profound validation of Paul Vitanyi’s foundational thesis that induction is compression [16]. For decades, Solomonoff induction was relegated to theoretical beauty, often deemed too idealized to inform practice. Today, as large models exhibit zero-shot generalization and run algorithms in-context, they offer compelling practical evidence for Vitanyi’s insight. Algorithmic probability and Kolmogorov complexity have shifted from abstract ideals to becoming a theoretical compass for understanding, interpreting, and scaling the next generation of (more) universal machines and their limits.

## Figures and Tables

**Table 1:** Overview of the main works surveyed in this paper.

Reference	Setting	Key Results/Implications
Ortega et al. [4]	Meta-learning theory	Log-loss minimization yields amortized Bayesian predictors.
Mikulik et al. [12]	Empirical confirmation of theory	Meta-trained predictors and policies match exact Bayes-optimal solutions.
Genewein et al. [8]	Non-stationary sources (PTW)	LSTMs match exact Bayesian inference (PTW algorithm) on piecewise-stationary sources with unobserved switching points.
Grau-Moya et al. [7]	Variable-order Markov sources (VoMs) & Solomonoff	Transformers match Bayes-optimal performance on VoMs (CTW); meta-learning can theoretically reach universal prediction.
Delétang et al. [9]	Language modeling is compression	LLMs trained on text compress images/audio better than domain-specific compressors.
Genewein et al. [5]	In-context learning theory	In-context learning is a necessary feature of Bayesian predictors (meta-trained nets).
Ruoss et al. [11]	Amortized chess engine	Amortization of complex algorithm; emergent planning.
Catt et al. [13]	Self-predictive agent	Formal bridge from Solomonoff prediction to AIXI-optimal action by letting the predictor do the heavy lifting.

**Table 2:** Compression rates (compressed size/raw size, in %; lower is better; best result per column in bold) across three data modalities, comparing text-trained LLMs against general-purpose and domain-specific compressors (data from Delétang et al. [9]). Adjusted rates account for model size via a two-part code, whereas raw rates measure only the compression rate of the data (1 GB in all cases). Billion-parameter Transformers trained only on text compress data of all modalities very well, suggesting that these models can learn and exploit (in-context) generally useful (algorithmic) patterns. This is not true for smaller Transformers, which compress text well but fail to transfer this capability to other modalities. All results use 2048-byte chunks matching the LLM context window; unchunked classical compressor results are reported in the original paper. Adjusted compression rates unsurprisingly show that, despite their strong raw compression rates, billion-parameter models do not constitute practical compressors for 1 GB files.

	Raw Rate (%)			Adjusted Rate (%)		
Compressor	enwik9	ImageNet	LibriSpeech	enwik9	ImageNet	LibriSpeech
gzip	48.1	68.6	38.5	48.1	68.6	38.5
LZMA2	50.0	62.4	38.2	50.0	62.4	38.2
PNG	80.6	61.7	37.6	80.6	61.7	37.6
FLAC	88.9	60.9	30.3	88.9	**60.9**	**30.3**
Transformer 200K	30.9	194.0	146.6	30.9	194.0	146.6
Transformer 800K	21.7	185.1	131.1	21.9	185.3	131.3
Transformer 3.2M	17.0	215.8	228.2	**17.7**	216.5	228.9
Llama 2 (7B)	8.9	53.4	23.1	1408.9	1453.4	1423.1
Chinchilla 1B	11.3	62.2	24.9	211.3	262.2	224.9
Chinchilla 7B	10.2	54.7	23.6	1410.2	1454.7	1423.6
Chinchilla 70B	**8.3**	**48.0**	**21.0**	14,008.3	14,048.0	14,021.0

**Table 3:** Amortized planning via supervised learning on chess (data from Ruoss et al. [11]). Transformers trained purely via log-loss minimization on Stockfish 16 value annotations of 10 M games (530 M board states), without any explicit search at test time. Baselines include AlphaZero and Leela Chess Zero (Lc0) variants with and without Monte Carlo tree search (MCTS). Note that different models use different training paradigms (supervised learning, self-supervised learning, and reinforcement learning) and different input formats (full-game PGN string vs. board-state FEN string), which limits direct comparability somewhat; see Ruoss et al. [11] for full details. Methods with a check mark in the ‘Search’ column use explicit search at test time and are shown for comparison.

Agent	Train	Search	Tournament Elo	Puzzle Acc. (%)
9 M Transformer	SL		2025(±18)	88.9
136 M Transformer	SL		2259(±16)	94.5
270 M Transformer	SL		2299(±15)	95.4
GPT-3.5-turbo-instruct	SSL		—	66.5
AlphaZero (policy only)	RL		1777(±25)	56.1
AlphaZero (value only)	RL		1992(±19)	82.0
AlphaZero (400 MCTS sim.)	RL	✓	2470(±16)	95.6
Lc0 (policy only)	RL		2292(±16)	88.6
Lc0 (value only)	RL		2418(±16)	95.9
Lc0 (400 MCTS sim.)	RL	✓	2858(±20)	99.6
Stockfish 16 (50 ms/move)	SL	✓	2711(±18)	99.8
Stockfish 16 (1.5 s/board)	SL	✓	2935(±23)	100.0

## Data Availability

No new data were created or analyzed in this study.
